# KBG syndrome mimicking genetic generalized epilepsy

**DOI:** 10.1016/j.ebr.2022.100545

**Published:** 2022-04-20

**Authors:** M.J. Murphy, N. McSweeney, G.L. Cavalleri, M.T. Greally, K.A. Benson, D.J. Costello

**Affiliations:** a Epilepsy Service, Department of Neurology, Cork University Hospital, Ireland; b College of Medicine and Health, University College Cork, Ireland; c Paediatric Neurology Service, Cork University Hospital, Ireland; d FutureNeuro SFI Research Centre for Chronic and Rare Neurological Diseases Hosted in RCSI, Dublin 2, Ireland; e The School of Pharmacy and Biomolecular Science, The Royal College of Surgeons in Ireland, Dublin, Ireland

## Abstract

• Several conditions may mimic Genetic Generalized Epilepsy GGE.
• GGE is less frequently misdiagnosed compared to other subtypes of epilepsy.
• KBG syndrome is a rare autosomal dominant condition.
• KBG syndrome may mimic GGE.

## Introduction

KBG syndrome is a rare autosomal dominant disorder characterised by short stature, craniofacial dysmorphism and other developmental skeletal and dental anomalies such as macrodontia [1]. The acronym KBG was chosen to represent the initial of the surnames of the three original families described and epileptic seizures are a common feature [1], [2], [3]. In addition shyness, anxiety, autistic spectrum disorders and hearing loss have all been reported and most affected patients exhibit developmental delay and intellectual disability [1], [4], [5].

Although considered polygenic in nature, Genetic Generalized Epilepsy (GGE) typically occurs sporadically [6]. Early descriptions reported high concordance rates among monozygotic twins [7], [8]. Descriptive reports highlighted consistent clinical and electroencephalographic (EEG) similarities in twin pairs. Because the clinical and EEG phenotypes are often striking and collectively pathognomonic, GGE is less frequently misdiagnosed compared to other subtypes of epilepsy. Nonetheless, GGE can be erroneously misdiagnosed in *Glut-1* deficiency, *CHD-2* mutations and focal epilepsy with a midline dipole [9], [10]. We report a pair of monozygotic twins who were initially diagnosed with GGE on clinical and EEG grounds. Whole exome trio testing was undertaken in their teenage years when their epilepsy proved drug-resistant and atypical clinical features became more prominent. Mutations in the *ANKRD11* gene confirmed a diagnosis of KBG syndrome. We report that mutations in the *ANKRD11* gene may produce a clinical syndrome that closely simulates sporadic GGE.

## Case report

### Twin 1

**Fig. 1:**
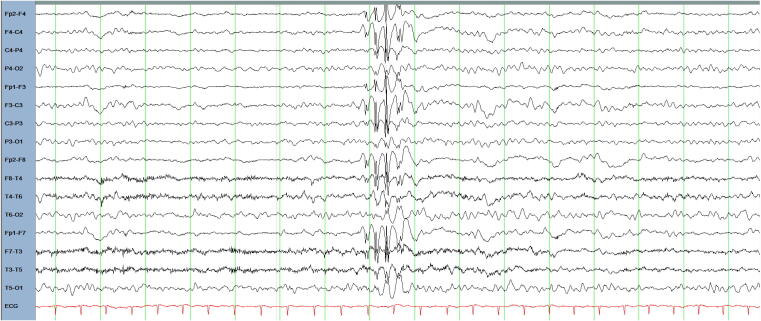
EEG of twin 1 age 16, bipolar montage in the awake state demonstrating a generalized fast spike-and-wave.

We report on a 20-year-old right-handed woman who was the product of a monozygotic twin pregnancy born by caesarean section delivery at 38 weeks of gestation with a birth weight of 2.0 kg. She was iron deficient at birth and spent some days in the neonatal intensive care unit (NICU) before being discharged home. She did not experience febrile convulsions, traumatic brain injury or central nervous system infections. Over time she was found to have a mild global learning disability requiring educational supports while attending mainstream education. She was noted to be very shy and reticent. Her family reported that she was quite timid and solitary. She displayed certain obsessions such as nail filing and hand washing. Her first seizure was a generalized tonic clonic seizure by description and occurred at age 11. Initially, there were 5 generalized tonic-clonic seizures per year on average, with 15 in total by the age of 13. A period of seizure freedom occurred from age 13 to 17 occurred after commencing zonisamide. There was no history of absence seizures or myoclonic seizures. Repeat EEGs demonstrated brief bursts of 3–4 Hz generalized spike-and-slow wave bursts of 1 second duration with maximal spike amplitudes over frontal regions (Fig. 1). Epilepsy protocol magnetic resonance imaging of her brain was normal. Her seizures proved difficult to control from age 17 when generalized tonic-clonic seizures recurred reaching a frequency of up to four seizures per month, despite trials of zonisamide, lamotrigine, levetiracetam and clobazam. A trial of topiramate in combination with zonisamide led to a marked reduction in seizure frequency, reducing to two generalized onset tonic clonic seizures in the year following treatment initiation.

At age 17, her clinical examination by a clinical geneticist revealed a height of 158 cm (<25th centile) and a head circumference of 55.5 cm (>50th centile). She had a triangular facial shape, high smooth hairline, bushy eyebrows, long palpebral fissures, high nasal bridge, small alae nasi, low hanging columella, short philtrum, thin upper lip, prominent chin, low-set ears with mild posterior rotation, mild left 5th digit clinodactyly, short digits and short and faint thenar creases on both palms. She had mild facial asymmetry with the left eye slightly smaller than the right.

### Twin 2

**Fig. 2:**
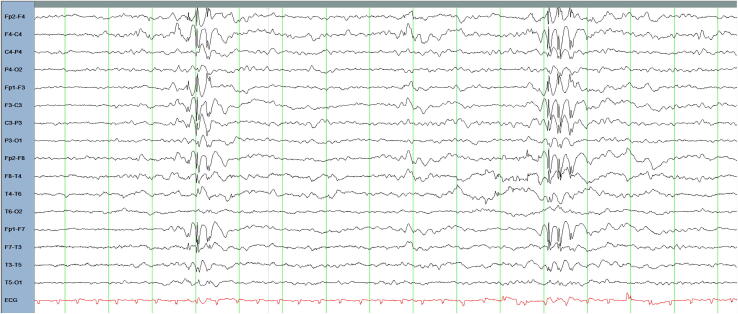
EEG of twin 2 age 16, bipolar montage in the awake state demonstrating a generalized fast spike and wave discharges.

Twin 1′s monozygotic twin sister first presented to adult neurology services at age 16. She suffered *in utero* from twin-twin transfusion syndrome and was delivered via caesarean section owing to breech presentation. She was born at a birthweight of 3.03 kg and spent some days in the neonatal intensive care unit (NICU) prior to discharge home. There were no early post-natal complications, and she has no personal history of febrile convulsions, traumatic brain injury or central nervous system infections. She was also noted to be shy, timid and solitary. At age 12, she presented in status epilepticus with repetitive generalized onset tonic clonic seizures. Additionally, she experienced recurrent episodes of absence status epilepticus manifesting with confusion and psychomotor slowing which were difficult to quantify. Between her first observed seizure at age 12 to transition to adult services at age 16 she experienced 6 generalized onset tonic clonic seizures. She had no history of myoclonic seizures. Previous trials with levetiracetam, lamotrigine and topiramate were poorly tolerated. A subsequent trial of topiramate was associated with sustained seizure freedom. After two breakthrough generalized tonic clonic seizures and one generalized absence seizure at age 17, the dose of topiramate was increased, leading to sustained freedom from generalized tonic clonic seizures in the following years. EEG revealed brief bursts of generalized 3–4 Hz spike-and-slow wave activity of 1 second duration (Fig. 2). Her brain imaging was normal.

At age 17 her clinical examination revealed height 159.5 cm (25th centile) and head circumference 54.5 cm (50th centile). Dysmorphic findings included a triangular face, high and irregular anterior hairline (higher on the right than the left), bushy eyebrows, long palpebral fissures, high nasal bridge, small alae nasi, low hanging columella, short philtrum, thin upper lip, pointed chin, low-set ears with mild posterior rotation, bilateral mild 5th digit clinodactyly, short digits, and thin and faint thenar creases on both palms. Both patients had early loss of deciduous teeth with subsequent dental crowding of permanent teeth. Macrodontia was not a prominent feature in either patient.

As a result of the distinct clinical phenotype in a monozygotic twin pair, trio whole exome sequencing was undertaken at age 17, of both probands and both parents, as part of a research project for which ethical approval was granted and full informed consent gained. No prior genetic testing had been performed. This delay to genomic testing was multifactorial as dysmorphic features were subtle initially, becoming more pronounced in teenage years and only truly fully appreciated by detailed examination for dysmorphic features by a clinical geneticist, but also due to resource constraints in terms of genomic testing during that time period in Ireland.

## Results

The whole exome sequencing revealed a *de novo* heterozygous variant, NM_013275.6:c.7571A > G;p.Glu2524Gly, in *ANKRD11* in both women [11]. The variant is absent from control sequences (Genome Aggregation Database (gnomAD) [12]) and no alternative plausible variants or known mutations were identified as competing possibilities in these patients. Multiple *in silico* prediction tools predicted a damaging effect on the resulting protein, with the variant classed as likely pathogenic according to American College of Medical Genetics and Genomics criteria. These results were discussed at the research group epilepsy genetics multidisciplinary team meeting and a clinical diagnosis of KBG syndrome was made. These results were replicated by an independent, accredited clinical testing laboratory on a second blood sample (CeGaT GmbH, Tübingen, Germany), including a concordant interpretation of the pathogenicity of the mutation.

## Discussion

The KBG phenotype includes generalized and focal seizures in addition to EEG abnormalities without clinically evident seizures [1]. Overall about half of patients with KBG syndrome have been found to have EEG abnormalities [1], [13]. Various seizure subtypes have been reported with tonic-clonic being the predominant seizure type described in the literature [1]. Reported cases include generalized and focal seizures and age of presentation varies from infancy to teenage years [1]. In most cases, as in ours, there is a therapeutic response to antiseizure medications, however drug-resistant cases have been reported [1], [14]. In our case, both twins had difficulty with frequent seizures prior to the initiation of topiramate which led to good seizure control in both cases. An early-onset severe phenotype which presents with infantile spasms has also been described and phenotypically there may be a clinical overlap with Glut-1 deficiency in how some cases present especially in early seizure-onset cases, with developmental delay [1].

In addition to Glut-1 deficiency several other conditions have been reported to have presented as GGE mimics or phenocopies such as Dravet syndrome, neuronal ceroid-lipofuscinoses type 2, late infantile GM2-gangliosidosis, myoclonic epilepsy with ragged-red fibers, Leigh syndrome, *CHD-2* mutations and focal epilepsy with a midline dipole [10]. Clinical features such as antiseizure drug resistance, focal neurological signs, dysmorphic features, atypical clinical and EEG features should prompt a revised search for alternative diagnoses [10]. Among the generalized epilepsies, close attention to the clinical and EEG characteristics are key to avoid a misdiagnosis of GGE [10].

## Conclusions

We report an identical twin pair presenting with a monogenic epilepsy syndrome closely simulating GGE due to mutations in the *ANKRD11* gene that is classically associated with the KGB syndrome. We describe novel findings of the KBG syndrome including subtle features of dysmorphism that should be considered among other conditions which may present as a GGE mimic.

## Ethical statement

Written informed consent was gained for inclusion in this case report.

## Declaration of Competing Interest

The authors declare that they have no known competing financial interests or personal relationships that could have appeared to influence the work reported in this paper.
